# Impact of coronavirus disease 2019 (COVID-19) pandemic isolation measures on the rate of non–COVID-19 infections in hematology patients

**DOI:** 10.1017/ice.2020.1279

**Published:** 2020-10-20

**Authors:** Jemima H. Miller, Stephen S. Opat, Jake Shortt, Despina Kotsanas, Claire Dendle, Maryza Graham

**Affiliations:** 1 Department of Hematology, Monash Health, Melbourne, Victoria, Australia; 2 Monash University School of Clinical Sciences, Monash Health, Victoria, Australia; 3 Department of Infectious Diseases, Monash Health, Melbourne, Victoria, Australia; 4 Department of Microbiology, Monash Pathology, Monash Health, Victoria, Australia; 5 Microbiological Diagnostic Unit Public Health Laboratory, The Peter Doherty Institute for Infection and Immunity, The University of Melbourne, Victoria, Australia

Laws concerning social distancing and restrictions on public life have been implemented globally during the COVID-19 pandemic. In turn, hospitals have changed their practice, from reduced visitation hours to stronger emphasis on hand hygiene. It would not be surprising, therefore, if transmission of non–COVID-19 infections were to decrease during this time, and data have started to emerge to support reduced incidence.^1^

In this study, we aimed to assess whether the incidence of non–COVID-19 infections had changed in a population of hematology patients while they have been subject to changes in infection control practices during the COVID-19 pandemic (Appendix 1 online). The hematology patient cohort was selected for the study population because they are often more susceptible to infections (from underlying disease or treatment), which, in turn, can result in higher rates of morbidity and mortality.^2^ The outcomes of this study will be used to inform continuation of isolation and infection control measures as the pandemic continues to evolve and afterward.

## Methods

Monash Health is a large, tertiary-care health network in southeastern Australia with a 32-bed hematology inpatient service. Hematology inpatients admitted between January 24 and May 23 (inclusive) for 2019 and 2020 were included in the study, a 4-month period from when the first patient with COVID-19 in Australia was admitted to Monash Health (January 24, 2020) and the equivalent dates the year prior to minimize effects of seasonal variation. Typical presentations for hospital admission included febrile neutropenia and elective inpatient chemotherapy.

All respiratory pathogen polymerase chain reaction (PCR), fecal pathogen PCR, and blood culture results from the cohorts were collated, and the proportion and breakdown of positive results were analyzed. Multiplex PCR assays (AusDiagnostics, Mascot, Australia) included targets for pathogens (Appendix 2 online). The rates of infection for each year were compared using the χ^2^ test or the Fisher exact as required. Statistical significance was set at *P* < .05. Statistical analyses were performed using Stata version 12.1 software (StataCorp LP, College Station, TX).

Any organism identified more than once in an individual patient within 7 days was considered a duplicate result. Regarding blood cultures, some organisms (listed in Appendix 2 online) were defined as contaminants if they were isolated in a single culture, but they were defined as pathogens if they were isolated in >1 culture within 7 days in the same patient.

## Results

Overall, 1,598 patients were admitted in the 2019 date range and 1,488 were admitted in 2020. The difference in infection rates was statistically significant for respiratory PCR (20.27% vs 10.90%; *P* = .01) but not for fecal PCR (8.00% vs 6.76%; *P* = 1.00) or blood cultures (3.27% vs 3.41%; *P* = .88) (Table 1).

**Table 1.** Breakdown of Microbiology Results by Type

Variable	Year		*P* Value
	2019	2020	
No. of hematology admissions	1,598	1,488	
Positive respiratory PCR results as a percentage of total respiratory PCR samples taken from hematology patient cohort	29 of 143 (20.27)	29 of 266 (10.90)	.01
Human metapneumovirus	2	0	
Influenza A/B virus	9	5	
Parainfluenza virus-1/-2/-3/-4	6	0	
Respiratory syncytial virus	2	3	
Rhinovirus/enterovirus	10	21	.74
Positive fecal PCR results as a percentage of total fecal PCR samples taken from hematology patient cohort	6 of 75 (8.00)	5 of 74 (6.76)	.77
*Campylobacter* spp	0	1	
*Clostridium difficile*	4	1	
*Giardia intestinalis*	1	1	
Norovirus	1	1	
*Salmonella* spp	0	1	
Positive blood cultures as a % of total blood cultures taken from hematology patient cohort	27 of 826 (3.27)	28 of 822 (3.41)	.88
Gram-positive bacteria^a^	**10**	**13**	.52
*Enterococcus faecium*	7	4	
*Staphylococcus aureus*	0	3	
*Staphylococcus* spp (coagulase negative)	1	3	
Other gram-positive bacteria	2	3	
Gram-negative bacteria^a^	**19**	**19**	.99
*Escherichia coli*	6	10	
*Klebsiella* spp	3	7	
*Pseudomonas aeruginosa*	3	0	
*Enterobacter cloacae* complex	2	0	
Other gram-negative bacteria	5	2	

Note. PCR, polymerase chain reaction.

a Includes cultures in which >1 organism was isolated.

Respiratory PCR virus swabs were procured in patients with respiratory infective symptoms and/or fever, and asymptomatic patients were not routinely tested. In 2019, 22 of the swabs were taken in the first 48 hours of presentation, and 24 were taken in 2020. The incidence of respiratory virus infection was lower in 2020 with 29 of 143 (20.27%) positive tests in 2019 compared to 29 of 266 (10.90%) in 2020 (*P* = .01). No cases of COVID-19 were identified. The incidence of all respiratory viruses was lower in 2020 except for respiratory syncytial virus and rhinovirus/enterovirus (Table 1).

There was no significant difference in the incidence of fecal pathogens, with 6 of 75 positive tests (8.00%) in 2019 compared to 5 of 74 (6.76%) in 2020 (*P* = .77). The rates of positive blood cultures were similar, with 27 of 826 positive isolates (3.27%) in 2019 compared with 28 of 822 (3.41%) in 2020, *P* = .88. Gram-negative bacilli were isolated in 19 positive blood cultures in both years. Excluding skin flora isolates, gram-positive organisms were isolated in 8 blood cultures in 2019 and 7 in 2020.

## Discussion

The percentage of positive respiratory PCR results was significantly lower in 2020 than 2019, when the hospital implemented changes in infection control practices and visitor restrictions during the COVID-19 pandemic (Appendix 1 online). The notable increase in the rates of rhinovirus/enterovirus in 2020, however, may be due to reduced activity of alcohol-based hand sanitizers on nonenveloped viruses.^3^

Blood cultures and fecal PCR results did not show statistically different rates of infection; these were mostly commensals or infections less affected by droplet precaution measures implemented during the pandemic.

Although similar numbers of blood cultures and fecal PCR samples were taken each year, we attribute the increased number of respiratory PCR samples taken in 2020 to heightened awareness for testing during the pandemic. Greater than 75% of all positive respiratory PCR samples were taken within 48 hours of admission, and although the results are unlikely to solely reflect community exposure (ie, our hematology patients frequently attend day treatment centers and other hospital services), a limitation is the difficulty in accounting for the expected reduction of circulating non–COVID-19 respiratory viruses in the community due to government restrictions and public behavior.

In hematology patients, viral infections are an important cause of morbidity and mortality.^4,5^ Therefore, increased awareness and utilization of infection control measures is vital for reducing rates of infection. Although our study was retrospective, we found a reduced rate of viral respiratory infection when stricter measures were in place at our hospital. This is a useful indication of their effectiveness, and incorporation into general hematology infection control can be considered. Further studies are warranted to assess the extent and duration of the impacts resulting from increased use of hand-sanitizer, limiting ward visitation, and social distancing on reducing infections within the hematology patient population.

## Appendix 1. Restrictions during the COVID-19 Pandemic Within the Hospital Network and the State From January 24 to May 23, 2020

**Table:**

Date	Monash Health	State of Victoria, Australia
January 24, 2020	First COVID positive patient in Australia is admitted at Monash Health.	
February 10, 2020	Hand hygiene posters produced in the 10 non-English languages most commonly spoken by patients	
February 21, 2020	Surgical masks provided in the ED for all patients with flu-like symptoms	
March 4, 2020	Campaign to refresh PPE knowledge launched including videos, online and in-person modules	
March 12, 2020	World Health Organization (WHO) names the COVID-19 crisis a pandemic	
March 15, 2020	Visitors limited to 2 per patient during a specified time period on the hematology ward	International travelers must quarantine on arrival for 2 weeks.
March 16, 2020		Gatherings of >500 people are banned.
March 18, 2020	Staff-only testing clinics open	
March 19, 2020	Campaign launched to focus on the WHO ‘Five Moments of Hand Hygiene’	
March 20, 2020	Most volunteers told to stop coming to the hospital	Australian border is closed to nonresidents.
March 23, 2020	Hematology ward closed to all visitors; staff must work from home where able.	
March 24, 2020		Lockdown starts, people must not leave home except for essential purposes. International and domestic travel is severely restricted.
March 29, 2020	Other wards only allow 1 visitor for 1 h per day (the hematology ward remains closed). All visitors screened on entry.	Gatherings are limited to 2 people.
March 30, 2020	Hematology day treatment unit is moved off the ward to a separate building.	
April 9, 2020	All visitors and staff have body temperatures checked on entry to the building.	
April 12, 2020	Staff encouraged to wear scrubs while at work.	
April 26, 2020		*COVIDSafe* app is released.
April 29, 2020	Asymptomatic staff encouraged to get tested at new clinics opened.	
May 8, 2020	Hand hygiene audit targets achieved for March and April.	

## Appendix 2

The respiratory pathogens 12 assay tests for the following organisms:

1. Adenovirus
2. *Bordetella pertussis*
3. Human metapneumovirus
4. Influenza A virus
5. Influenza B virus
6. Parainfluenza viruses -1, -2, -3, and -4
7. Picornavirus (rhinovirus/enterovirus)
8. Respiratory syncytial virus
9. Severe acute respiratory coronavirus virus 2 (SARS-CoV-2)

The fecal pathogens B assay tests for the following organisms:

1. Adenovirus
2. Astrovirus
3. *C. difficile*
4. *Campylobacter jejuni/coli/doyeli*
5. *Cryptosporidium spp*
6. *Entamoeba histolytica*
7. *Giardia intestinalis*
8. Norovirus
9. Rotavirus
10. *Salmonella spp*
11. Sapovirus
12. Shiga toxin 1 and 2
13. *Shigella spp*
14. *Yersinia spp*

The Bactec FX (BD Diagnostics) blood culture system was used. The following organisms were defined as contaminants if isolated in a single positive culture:

1. *Coagulase-negative Staphylococcus*
2. *Bacillus spp*
3. *Propionibacterium/Cutibacterium spp*
4. *Viridans streptococci*
5. *Corynebacterium spp*
6. *Kocuria spp*
7. *Micrococcus spp*

In cases of a repeat positive blood culture within 7 days with the same organism, the isolate was defined as pathogen.
